# Xeno-free protocol for GMP-compliant manufacturing of human fetal pancreas-derived mesenchymal stem cells

**DOI:** 10.1186/s13287-022-02946-5

**Published:** 2022-06-21

**Authors:** Zahra Jabbarpour, Sajjad Aghayan, Babak Arjmand, Khadijeh Fallahzadeh, Sepideh Alavi-Moghadam, Bagher Larijani, Hamid Reza Aghayan

**Affiliations:** 1 Gene Therapy Research Center, Digestive Disease Research Institute, Tehran University of Medical Sciences, Tehran, Iran; 2 Cell Therapy and Regenerative Medicine Research Center, Endocrinology and Metabolism Molecular-Cellular Sciences Institute, Tehran University of Medical Sciences, No 111, 19th Allay., North Kargar St., P.O.Box:14117-13137, Tehran, Iran; 3 Endocrinology and Metabolism Research Center, Endocrinology and Metabolism Clinical Sciences Institute, Tehran University of Medical Sciences, Tehran, Iran

**Keywords:** Cell therapy, Diabetes, Fetal pancreas, GMP, Mesenchymal stem cell, Xeno-free

## Abstract

**Background:**

Mesenchymal stem cells (MSCs) have been suggested as an appropriate source for diabetes cell-based therapies. The high proliferation and differentiation capacity of fetal MSCs and the role of fetal pancreatic-derived MSCs (FPMSCs) in islet generation make them good candidates for diabetes treatment. To manufacture clinical-grade MSCs, animal-free culture protocols are preferred. The current study aimed to establish a xeno-free/GMP-compliant protocol for FPMSCs manufacturing. The focus was on the effects of fetal bovine serum (FBS) replacement with pooled human serum (HS).

**Material and methods:**

FPMSCs were isolated and expanded from the pancreas of legally aborted fetuses with few modifications in our previously established protocol. The cells were expanded in two different culture media, including DMEM supplemented with 10% FBS or 10% pooled HS. A side-by-side comparison was made to evaluate the effect of each serum on proliferation rate, cell cycle, senescence, multi-lineage differentiation capacity, immunophenotype, and tumorigenesis of FPMSCs.

**Results:**

Flow cytometry analysis and three-lineage differentiation ability demonstrated that fibroblast-like cells obtained from primary culture had MSCs’ characteristics. The FPMSCs displayed similar morphology and CD markers expression in both sera. HS had a higher proliferative effect on FPMSCs than FBS. In FBS, the cells reached senescence earlier. In addition to normal karyotypes and anchorage-dependent growth, in vivo tumor formation was not seen.

**Conclusion:**

Our results demonstrated that HS was a better serum alternative than FBS for in vitro expansion of FPMSCs. Compared with FBS, HS increased FPMSCs’ proliferation rate and decreased their senescence. In conclusion, HS can effectively replace FBS for clinical-grade FPMSCs manufacturing.

**Graphical abstract:**

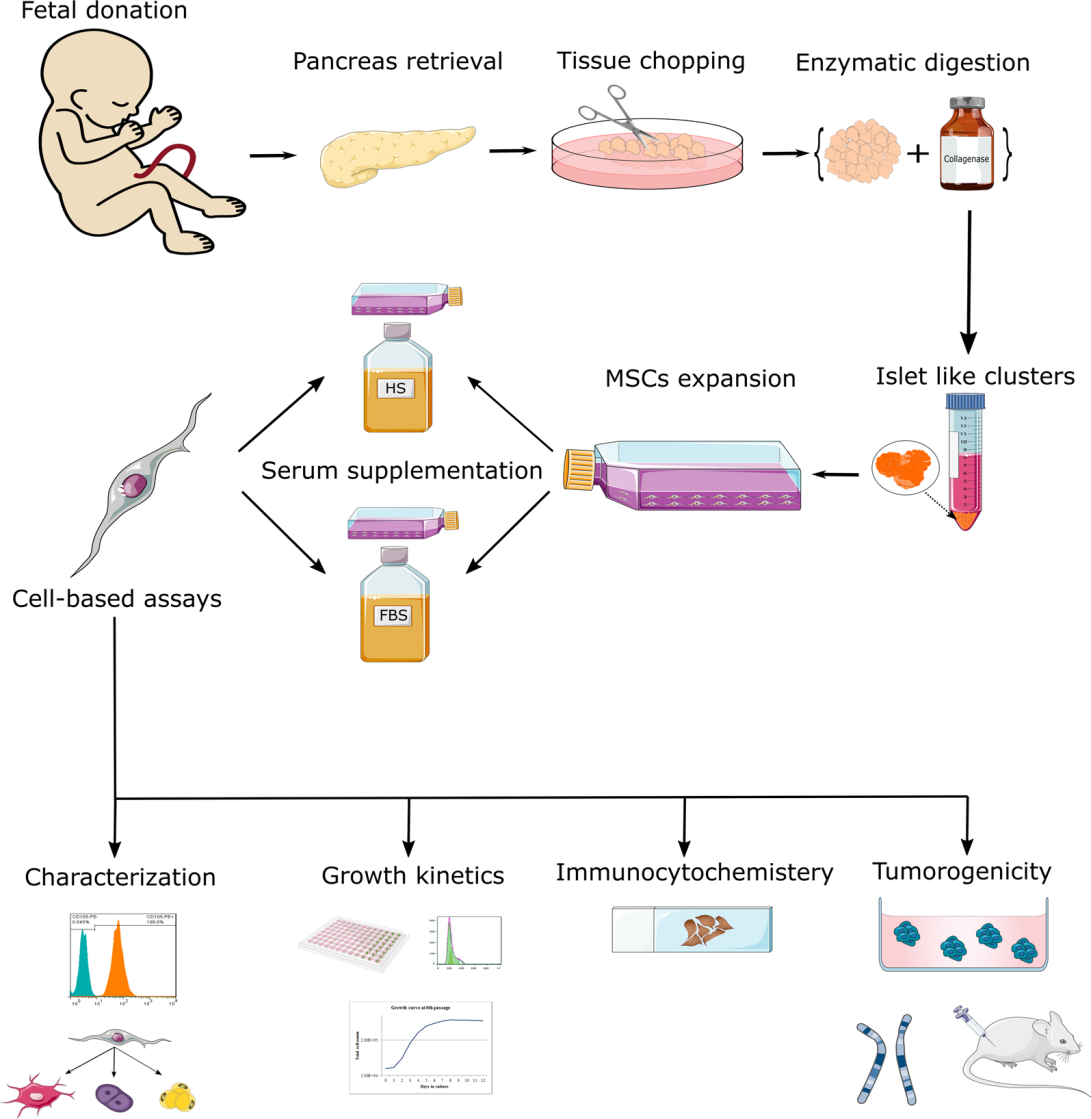

## Introduction

Type 1 diabetes mellitus (T1DM) is an autoimmune disease caused by the destruction of insulin-producing beta cells. The current treatment modalities aim to control diabetes and its complications effectively [1]. The socioeconomic impact of T1DM has motivated scientists to search for curative treatment. As a new curative approach, islet transplantation has several limitations: the scarcity of islets, transplant rejection, immunosuppression, difficulties related to islet isolation, cell loss during the separation and transplantation process, and high costs [2]. Therefore, finding alternative cell sources for regenerating or replacing damaged islets is crucial. In recent decades, stem cell-based therapies have raised hopes for treating incurable diseases such as diabetes [3]. Mesenchymal stem cells (MSCs) have received more attention due to their proliferation and differentiation capacity and low immunogenicity. MSCs can migrate to the pancreas, reduce apoptosis and residual beta cell mass loss, and stimulate pancreatic stem cells to proliferate and differentiate into endocrine cells [4, 5]. Despite similar phenotypes and the common mechanisms of tissue regeneration, the source of MSCs can play a crucial role in their therapeutic effects [6]. It has been suggested that tissue-matched MSCs may increase the efficacy of their regenerative effects [7]. Studies have shown that diversity in the microenvironment of MSCs and, subsequently, the expression of different genes lead to differences in their function and behavior [8]. It is suggested that MSCs isolated from each tissue may be a more appropriate source for regenerating the same tissue. For instance, studies have shown that pancreatic-derived MSCs can play a more supportive and regenerative role for pancreatic islets [9–11]. Fetal pancreatic-derived MSCs (FPMSCs) are highly proliferative cells that play a crucial role in fetal pancreas development [12]. In vivo studies have been conducted to evaluate the therapeutic effect of FPMSCs in diabetes. These studies showed that they could be engrafted, differentiated, and exhibited islet cell-like function in the animal model. Furthermore, they have less immunogenicity and higher differentiation and proliferation capacity than adult MSCs [13, 14]. In our recent study, we examined the impact of the three-dimensional co-culture of FPMSCs on human embryonic stem cell-derived pancreatic progenitors’ development. This study revealed higher expression of NGN3 and INSULIN in FPMSCs than bone marrow-derived MSCs group [10]. Before clinical application, FPMSCs should be expanded in vitro to make an appropriate dose. Optimal MSCs’ expansion requires a 10–20% serum in a culture medium that significantly enhances proliferation and differentiation [15]. The source of serum is one of the most important safety considerations in clinical-grade cell manufacturing. Fetal bovine serum (FBS) is widely used for MSCs’ expansion [16, 17]. The use of animal derivatives can lead to adverse immune reactions such as anaphylactic shock or cardiac arrhythmia and may transmit viral and bacterial infections [18]. Studies showed that xeno-free sera such as human serum (HS) or human platelet lysate (hPL) could be used for MSCs’ culture. Indeed, MSCs’ expansion for clinical application should be carried out according to current good manufacturing practices (cGMP) [19]. GMP covers all aspects of the manufacturing process, including raw material selection and qualification. It emphasizes that all materials that come in direct contact with cell therapy products should be of appropriate quality. Additionally, there are more specific considerations for animal-sourced products [20]. Hence, to increase the safety of cell therapy products, most regulatory agencies recommend omitting animal-derived materials in the cell manufacturing process [21–23]. Regarding the possible importance of MSCs in support of islet regeneration and considering the unique potential of FPMSCs in the treatment of diabetes, we aimed to optimize the FPMSCs’ expansion under xeno-free/GMP-compliant conditions.

## Materials and methods

## Isolation and expansion of FPMSCs

To procure the legally aborted fetus, written informed consent was obtained from parents according to the procedures approved by the Ethics Committee of Endocrinology and Metabolism Research Institute, Tehran University of Medical Sciences, Tehran, Iran (IRB code: EC-00264). In addition to routine donor evaluation, HIV, HBV, HCV, CMV, EBV, HTLV, Toxoplasma, and venereal diseases were examined in the mother’s blood sample. FPMSCs were isolated and expanded according to our previously described method with few modifications [24]. Briefly, the pancreas was aseptically harvested from the aborted fetus (14–18 weeks) by a midline laparotomy following the left subcostal extension. The harvested tissue was washed three times with PBS/EDTA buffer (CliniMACS, Miltenyi Biotec, Germany) containing 200 units/ml penicillin, 0.2 mg/ml streptomycin, and 0.5 µg/ml amphotericin B (antibiotic/antimycotic solution, Biowest, France), trimmed, minced in small fragments, and partially digested using animal-free collagenase CLSAFA/AF (Worthington, USA) at 37 °C for 10 min. The digestion process was terminated by adding an equal volume of cold PBS/EDTA and allowing fragments to settle on ice for 10 min. The small tissue fragments were washed with PBS/EDTA and centrifuged for 5 min at 300 × g. The pellet was resuspended in DMEM-LG supplemented with 15% FBS (Both from Biowest, France), seeded into a 25 cm^2^ culture flask, and incubated at 37 °C, 5% CO2, and 95% humidity. To deplete non-adherent cells, the culture medium was changed after 72 h, and cell expansion was continued up to 90% confluency (10–14 days). Then, adherent cells were harvested using TrypLE™ solution (Thermo Fisher, USA) and seeded into new culture flasks containing DMEM-LG + 10% FBS (1 to 4 ratio). At the third passage (P3), the cells were harvested, counted, and cryopreserved (10% DMSO + 90% culture medium (DMEM-LG + 10% FBS)) for future studies. All tissue processing steps and cell manufacturing were performed under aseptic conditions using a Class II biological safety cabinet located in a Grade B cleanroom. Samples from each batch were examined for identity, potency, sterility, mycoplasma, and endotoxin. To evaluate and compare the effects of different sera, fully characterized FPMSCs were thawed, washed, and resuspended in PBS/EDTA. Then, 3 × 10^3^ cells/cm^2^ were seeded into culture flasks containing DMEM-LG + 10% FBS (FBS group) or DMEM-LG + 10% HS (pooled AB serum, Biowest, France, (HS group). Before a side-by-side comparison, the cells were adapted to the new serum for three consecutive subcultures.

## Characterization of FPMSCs

To characterize FPMSCs, each cryopreserved batch was evaluated at the third passage (P3) for CD markers’ expression, morphology, and three-lineage differentiation capacity [25]. Then, fully characterized FPMSCs were used for the experiments.

### Flow cytometry analysis

At P3, FPMSCs (1 × 10^6^) were incubated for an hour in PBS containing BSA 3% (Atocel, Austria) at 4 °C. Then, the cells were labeled with specific conjugated antibodies or isotype-matched control (all from eBioscience, Inc., USA) against CD markers according to the manufacturer’s instructions for 45 minutes at 4 °C. The labeled cells were resuspended in FACS buffer (FACSCalibur^TM^, BD Biosciences, USA) after washing twice with PBS (Biowest, France). For FPMSCs characterization, CD11b-FITC, CD45-FITC, CD19-PE, CD34-PE, HLA-DR-PE, CD105-PE, CD73-PE, and CD90-PE were examined. Moreover, to evaluate the effect of serum supplementation on FPMSCs’ phenotype, CD105-PE, CD73-PE, CD90-PE, and HLA-DR-PE were assayed in each study group. Flow cytometry analysis was done by Saba Biomedical laboratory (Tehran, Iran)

### Multi‑lineage differentiation

StemPro® Osteogenesis, Adipogenesis, and Chondrogenesis Differentiation Kits were used to evaluate the differentiation capacity of FPMSCs (all from Invitrogen, USA). For the osteogenic differentiation study, FPMSCs were seeded into 4-well plates (1.5 × 10^4^ cells/well). After 21 days of incubation, the cells were fixed with 4% PFA (paraformaldehyde) for 20 minutes at room temperature (RT), and calcium deposition was stained with an Alizarin Red S solution (Sigma, USA). For adipogenic differentiation, 2.5 × 10^4^ cells/well were seeded in 4-well plates. After 14 days of culture, the cells were fixed, and the formation of lipid vacuoles in FPMSCs was examined using Oil Red O (Sigma, USA) staining. Chondrogenesis media was added to 2.5 × 10^5^ FPMSCs, and the suspension was centrifuged (200 × g/5 minutes) to evaluate chondrogenic differentiation. The supernatant was discarded without disturbing the pellet, and 0.5 ml of differentiation media was added. The tubes were incubated with loose caps in an upright position at 37 °C and 5% CO2. After 28 days, the cell nodules were fixed in 4% PFA overnight. The paraffin blocks were prepared from fixed nodules and then stained with Alcian blue to evaluate cartilage matrix formation (the Pathology Department of the Shariati Hospital, Tehran, Iran). FPMSCs from each group were examined for osteogenic and adipogenic differentiation capacity to assess the effect of serum supplementation. Images were taken using an inverted microscope (Eclipse TS100, Nikon, Japan).

## Growth kinetic

The proliferation rate and growth pattern of FPMSCs were evaluated by cell cycle analysis, population doubling time (PDT) calculation, and plotting the growth curve. For cell cycle analysis, 5 × 10^5^ FPMSCs were fixed in cold 70% ethanol (4 °C, 1 h). After washing twice with cold PBS (800 × g /5 min), the cell samples were sent to the flow cytometry department of Saba Biomedical Laboratory (Tehran, Iran). Further processing of the samples was done according to their protocols, and the percentage of FPMSCs in S + G2 phases was determined. To calculate the PDT, 7 × 10^3^ cells/well were seeded into 12-well culture plates. At 80% confluency, the cells were harvested and counted, and the PDT was calculated by this formula:

$${\text{PDT}} = \frac{{{\text{Culture duration}} \left( h \right) \times \log \left( 2 \right)}}{{\log \left( {\text{Final concentration}} \right) - \log \left( {\text{Initial concentration}} \right)}}$$

Cell expansion was continued for five consecutive passages (P3 to P8), and the mean of the PDT was calculated. FPMSCs were seeded at a density of 7 × 10^3^ cells/well in 12-well plates and were incubated at 37 °C and 5% CO2 to plot the growth curve. The cells were counted daily, and the mean numbers were used to draw the growth curve.

## Senescence assay

Senescence-associated β-galactosidase (SA-βG) assay was applied to evaluate FPMSCs’ senescence. The cells were seeded into 12-well plates at 4 × 10^4^ cells/well in different media and cultured up to 60% confluency. After fixation with 2% PFA and 0.2% glutaraldehyde, the cells were stained using X-gal (5-Bromo-4-chloro-3-indolyl β-D-galactopyranoside) staining kit (Sigma-Aldrich, USA) according to the manufacturer’s instruction. The presence of SA-βG (a blue precipitate in the cytoplasm) was investigated by an inverted microscope.

## Immunocytochemistry

To evaluate the effect of serum on FPMSCs’ protein expression, immunocytochemistry (ICC) was performed. The cells from each group were seeded into 4-well plates at 1 × 10^4^ cells/well. At 50–60% confluency, the cells were fixed with 4% PFA, washed three times in 0.1% PBS/Tween20 (PBST), permeabilized using PBS + 0.25% Triton X-100 (10 min /RT), and blocked by blocking buffer (10% goat serum and 1% Albumin in PBST) for 1 h at RT. Finally, according to the manufacturer’s instructions, the primary antibodies were diluted in dilution buffer (PBST + 1% Albumin) and added to each sample. For this experiment, anti-Nestin (14–9843-80), anti-Vimentin (14–9897-80), anti-Ki67 (14–5699-80), anti-HLA-ABC (14–9983-80), and Mouse IgG1 K Isotype Control (14–4714-81) antibodies were used (all from eBioscience, USA). The cells were incubated in primary antibodies for an hour at 37 °C. EnVision Detection Kit (Dako, Denmark) was used to visualize the antigen–antibody reaction. Briefly, the secondary antibody (goat-HRP anti-Rabbit/Mouse Ab) was added (incubation at 37 °C for 30 min). After washing with PBST, 50 µl of DAB chromogen solution and 1 ml substrate buffer were added (5–10 min, RT), and the cells were examined under an inverted microscope. To quantify the Ki67 expression, the number of stained cells was calculated in 10 different microscopic fields (20X objective) with ImageJ software (v1.53f) using the “Cell Counter” plugin (plugins → analysis → cell counter) [26].

## Tumorigenesis assay

Tumorigenesis potential of in vitro expanded FPMSCs was examined according to the recommendation of WHO technical report series No. 978 /61st report, 2013 [27].

### Karyotype analysis

FPMSCs (at different passages) were sent to the Genetics Laboratory of Sarem Hospital (Tehran, Iran) for G-banding karyotyping.

### Soft agar colony formation assay

The anchorage-independent growth ability of FPMSCs was examined by a soft agar colony formation assay using a previously published protocol [28]. Briefly, 1% sterile agar solution (UltraPure™ Agarose, 16500100, Thermo Fisher, USA) was mixed with 2X DMEM+20% FBS in a 1:1 ratio and added to a non-treated 6-well plate (base agar). To prepare top agar, three different numbers of FPMSCs (50, 20, and 10 × 10^4^ cells) at 6th and 10th passages were mixed with 2X DMEM+20% FBS and 6% sterile agarose and added to base agar. The plates were incubated in 5% CO2, 37 ºC, and 95% humidity for 21 days. Colony formation was examined under an inverted microscope after crystal violet staining (0.1% W⁄V). HEK 293T cells (donated by Sabz Biomedicals, Tehran, Iran) were used as a positive control.

### In vivo tumorigenicity assay

According to relevant ethical and institutional guidelines, the animal experiment was performed by the Gene Therapy Research Center (Digestive Disease Research Institute, Tehran University of Medical Sciences, Tehran, Iran). Briefly, 10 × 10^6^ viable cells were suspended in 0.1 ml PBS and injected intramuscularly into 10 athymic mice (4–7 weeks old). Considering the principle of “reduction” in animal experiments, we decided to perform in vivo assay only for HS-supplemented group (as the optimal protocol). The HEK 293T cells were used as a positive control. All animals were examined weekly (up to 16 weeks) for any evidence of nodule formation at the injection site. At the end of the experiment, all animals were euthanized and examined for gross and microscopic evidence of the proliferation of inoculated cells at the injection site and vital organs.

## Statistical analysis

The FPMSCs were isolated from three different donors, and all in vitro experiments were performed in triplicates. Paired T test was used to compare the quantitative data of each group. Results were reported as the mean ± SD and *P* value < 0.05 were considered significant.

## Results

### Isolation, expansion, and characterization of FPMSCs

**Fig. 1:**
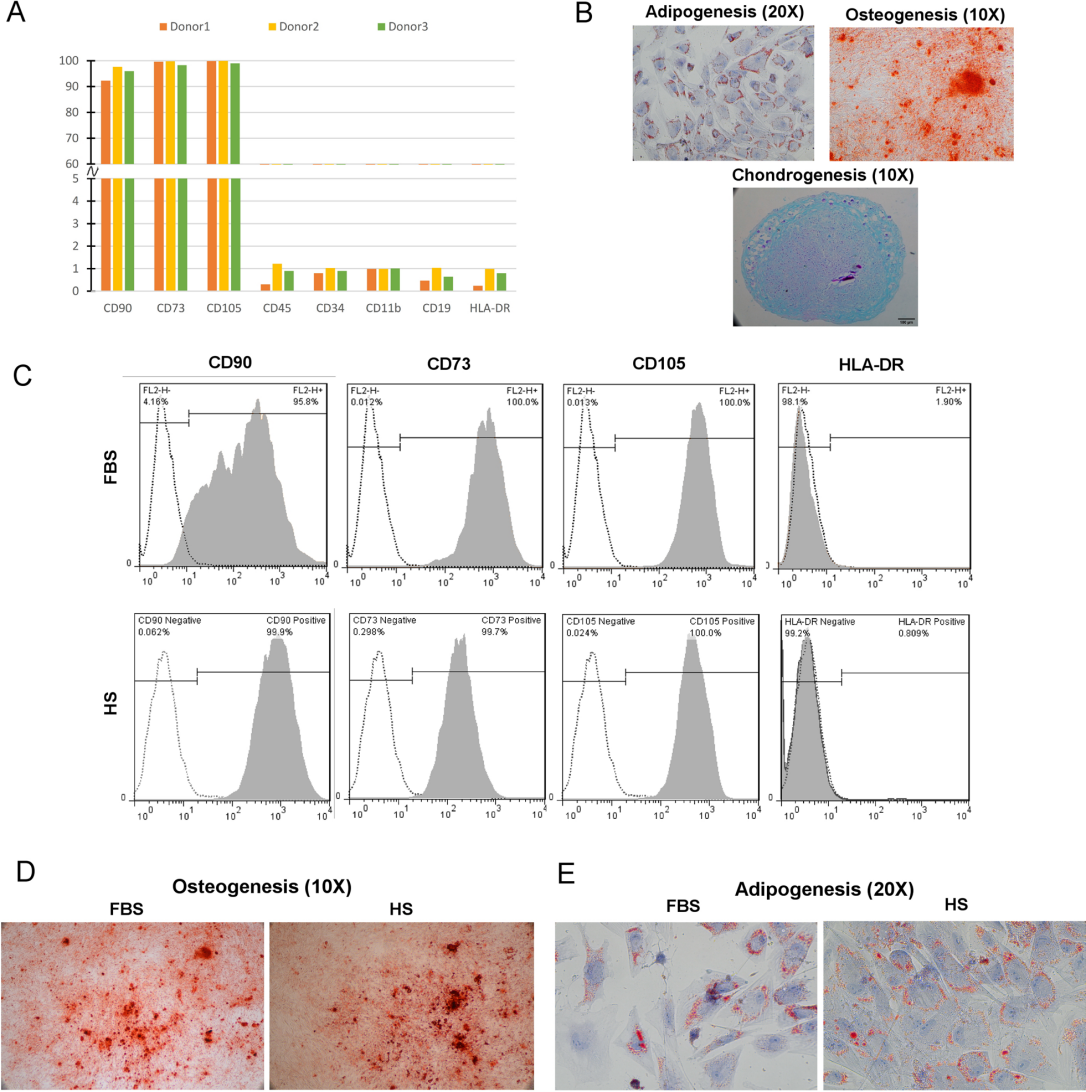
FPMSCs’ characterization at the third subculture: **A** CD marker expression of FPMSCs isolated from different donors, **B** three-lineage differentiation of FPMSCs. The expression of mesenchymal CD markers and HLA-DR in both study groups **C**. The effect of different sera on FPMSCs’ differentiation to osteocytes **D** and adipocytes **E**

FPMSCs were successfully isolated from three consecutive fetal pancreases. At P3, a homogenous population of spindle-shaped cells was seen. CD marker expression panel demonstrated that they express mesenchymal markers and lack hematopoietic markers and HLA-DR (Fig. 1A). Furthermore, they could differentiate into adipocytes, osteocytes, and chondrocytes using appropriate media (Fig. 1B).

### CD marker expression and differentiation capacity in different sera

**Fig. 2:**
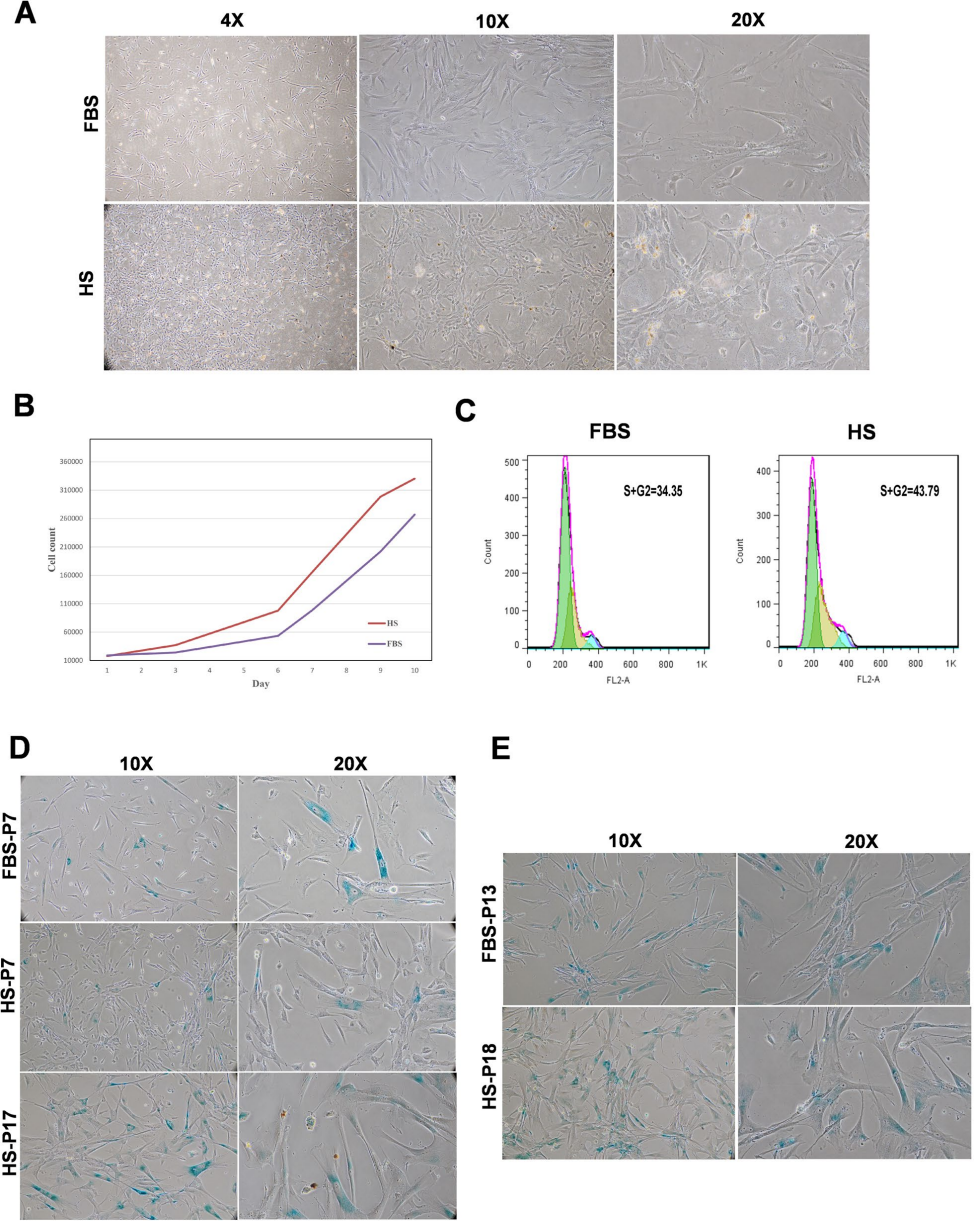
**A** FPMSCs’ morphology in different sera. **B** Growth curve of FPMSCs in HS- and FBS-supplemented media. **C** The results of cell cycle analysis. **D** and **E** SA-βG assay of FPMSCs from two batches

After three consecutive passages in FBS or HS, no changes were seen in the expression of PLMSCs’ CD markers (Fig. 1C) or their differentiation capacity to osteocytes (Fig. 1D) and adipocytes (Fig. 1E). In both groups, PLMSCs showed spindle-shaped morphology. However, the cells in the HS group were thinner and smaller than in the FBS group (Fig. 2A).

### Growth kinetic and senescence of FPMSCs in different sera

After several passages (P8), our results showed that the mean of PDT in the HS group (77.74 $\pm \hspace{0.17em}$22.52 h) was significantly (*P* < 0.0001) lower than in the FBS group (94.32 $\pm \hspace{0.17em}$24.48 h). In addition, the cell yield/cm^2^ of the culture area was 22,609.78 $(\pm \hspace{0.17em}$6397.84) in the HS group and 16,864 $(\pm \hspace{0.17em}$5993.90) in the FBS group (*P* < 0.0001). The growth curves showed that HS had increased the proliferation rate of FPMSCs (Fig. 2B). Cell cycle analysis revealed that the mean of FPMSCs in S + G2 phases in the HS group (32.98 $\pm \hspace{0.17em}$8.55) was significantly (*P* < 0.05) higher than in the FBS group (22.70 $\pm \hspace{0.17em}$4.22). Figure 2C illustrates the results of cell cycle analysis in one representative sample. The results of the SA-βG assay revealed that FBS accelerated the senescence of serially passaged FPMSCs. In one sample, we found early senescence of FPMSCs in FBS at P7, while they could be expanded in HS up to P17. In two other samples, the cells were proliferative in FBS up to P13 and P15 and in HS up to P18 and P20, respectively. Figure 2D, E illustrates the expression of SA-βG in each study group of two different PLMSC samples. 

### Proteins expression profile

**Fig. 3:**
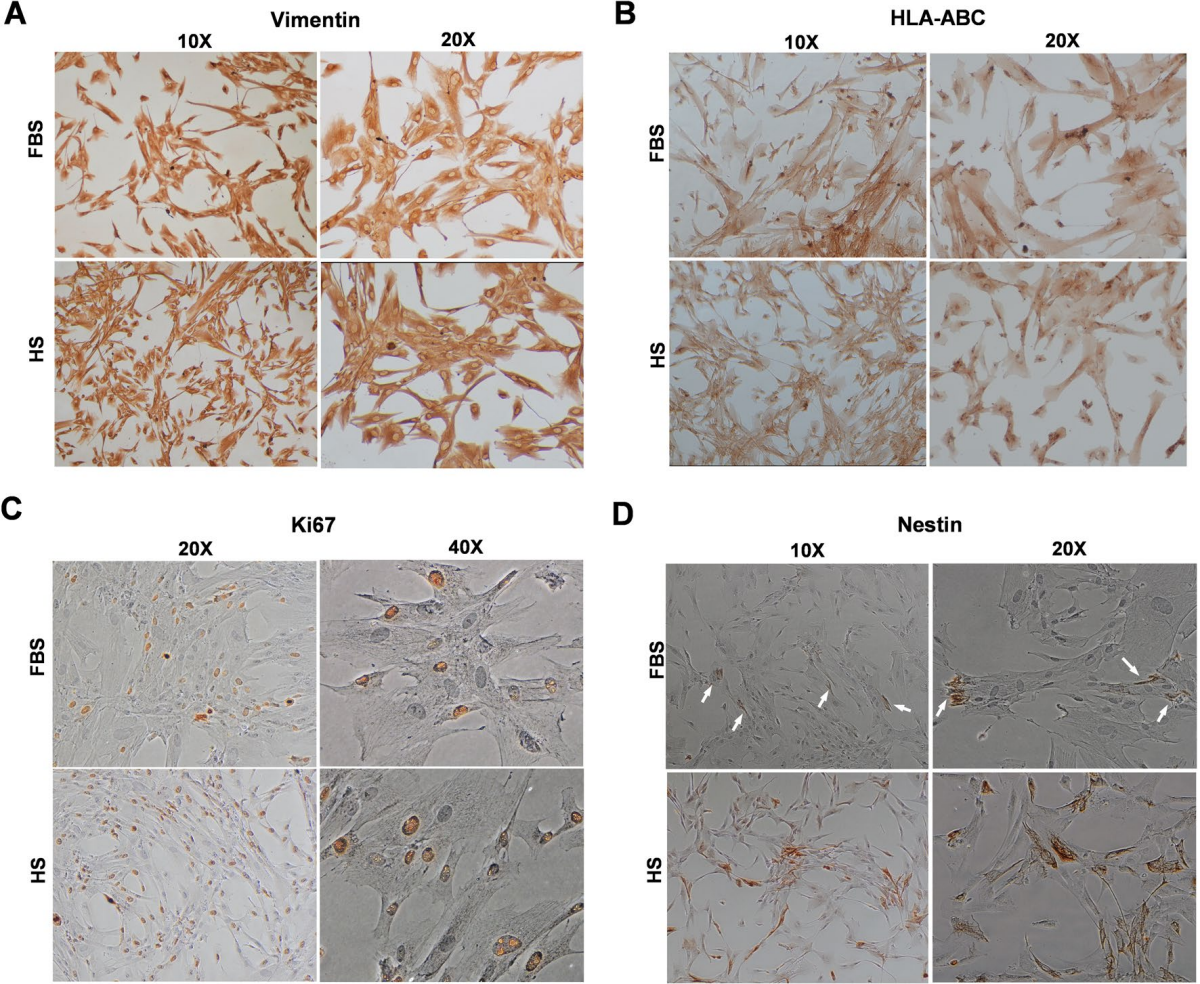
ICC assay of FPMSCs in both study groups. **A** Vimentin, **B** HLA-ABC, **C** Ki67, and **D** Nestin. The white arrows show the Nestin expression in FBS-cultured cells

ICC studies demonstrated that Vimentin (Fig. 3A) and HLA-ABC (Fig. 3B) were expressed equally in both study groups. The expression of Ki67 (Fig. 3C) and Nestin (Fig. 3D) was higher in the HS group. The mean of Ki67 expression was 54%$(\pm \hspace{0.17em}$8.04) in the HS group and 44%$(\pm \hspace{0.17em}$4.24) in the FBS group (*P* < 0.01).

### Tumorigenicity assay

**Fig. 4:**
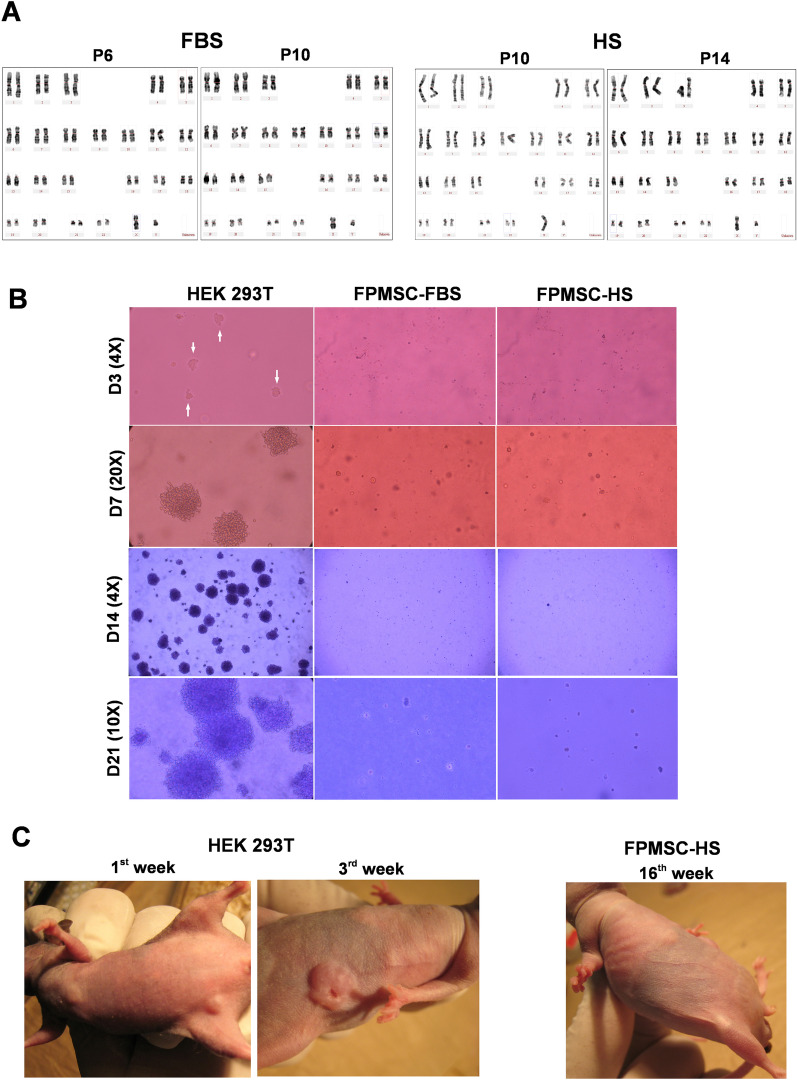
Tumorigenicity assay of FPMSCs in HS- and FBS-supplemented media. **A** G-banding karyotyping, **B** soft agar colony formation assay, **C** tumor formation in nude mice. The white arrows show early colony formation of HEK 293 T cells

G-banding karyotyping demonstrated chromosomal stability of in vitro expanded FPMSCs in both groups at different passages. All samples showed a normal 46XY karyotype (Fig. 4A). Figure 4B illustrates the results of soft agar colony formation assays in the HS and FBS group at the sixth passage. The assay revealed that HEK 293 T (positive control) could generate large colonies after 14 days. In contrast, no colony formation was seen in FPMSCs’ groups until the 28th day of culture. The same results were observed for FPMSCs in the 10th passage. Similarly, in vivo studies revealed that HS-cultured FPMSCs did not induce tumor formation in nude mice up to 4 months post-transplantation. In positive controls, nodule formation was seen after 1 week and was prominent in the second-week post-transplantation (Fig. 4C).

## Discussion

For decades, exogenous insulin injection has been suggested as the main treatment option for patients with T1DM. Despite regular insulin injections, fewer than 40% of patients can reach and maintain a stable euglycemic state. Recently, many researchers have focused on finding more efficient therapies for T1DM. The most promising approach is to restore beta cell mass or functionality using regenerative medicine technologies. Cell-based therapies and tissue engineering aim to improve the length and quality of patients’ life by regenerating, preserving, or enhancing beta cell functions [29]. In the last two decades, many protocols have been successfully developed to generate insulin-producing cells (IPCs) or islet organoids in vitro. Embryonic stem cells (ESCs) and MSCs have shown better outcomes among different cell types. In addition to IPCs’ generation, MSCs have been co-cultivated with ESCs to support islet organoid formation. Previous studies have shown that FPMSCs express different genes, proteins, and signaling pathway factors than other mesenchymal sources, suggesting them as a more suitable candidate for diabetes treatment [10]. In autoimmune and systemic diseases (like diabetes), the proliferation and differentiation capacity of autologous MSCs are significantly impaired. In such cases, allogeneic MSCs may have better therapeutic effects. Among different sources of allogeneic cells, priority should be given to those with higher potency, more safety, and lower immunogenicity. Fetal-derived MSC seems a suitable source for regenerative therapies because they are safer than ESCs and have higher potency and less immunogenicity than adult SCs [30, 31]. To prepare a therapeutic dose, MSCs should be expanded in vitro with the principles of GMP [32]. Along with other principles of GMP, careful selection of ancillary materials (AMs) is essential to assure the product’s safety and efficacy [33]. It is preferred to omit xenogeneic materials from the manufacturing process when applicable. The current study describes a xeno-free/GMP-compliant protocol for the in vitro expansion of FPMSCs. Our results demonstrated that FPMSCs were expanded efficiently using our xeno-free protocol. In addition, we could successfully modify our previous isolation protocol [24] using an animal-origin-free collagenase. FPMSCs’ characterization studies revealed no significant difference between FBS and HS groups. Both culture conditions yielded a pure population of FPMSCs, which lacked HLA-DR, expressed Vimentin, HLA-ABC, CD 90, CD105, and CD73, and were differentiated into osteocytes and adipocytes. The cells in both groups showed spindle shape morphology, but their size was smaller in the HS group (Fig. 2A). Some studies have shown that HS and FBS have similar effects on the differentiation potential of MSCs [34, 35]. However, few studies have indicated that HS increased the osteogenic differentiation of MSCs [19, 36]. Our differentiation studies aimed to evaluate the effect of serum supplementation on FPMSCs’ characteristics. To assess the effect of different sera on the differentiation capacity of MSCs, quantitative studies are needed. The growth curves demonstrated that FPMSCs proliferate more rapidly in HS-supplemented media. Similarly, cell cycle analysis revealed that the average of cells in S + G2 phases was significantly higher in the HS group (*P* < 0.05). Moreover, FPMSCs in the HS group showed higher expression of the Ki67 proliferation marker (*P* < 0.05). These findings imply that HS has a more proliferative effect on FPMSCs than FBS (Fig. 2C). From the 10th passage, FPMSCs in the HS group had a lattice growth pattern and were spindle-shaped until the 15th. In a similar study, ADMSCs cultured in HS showed a lattice growth pattern from the fourth passage with faster growth and smaller size than FBS [22]. Similarly, de Paula et al. reported that ADMSCs proliferated more rapidly in HS, and the cell density per unit area was higher than FBS [37]. In addition to the proliferation assays, we compared FPMSCs’ senescence during serial passages. The results of the SA-βG assay showed accelerated cellular senescence in FBS compared with the HS group. To sum up, the HS supplementation increases the yield of FPMSCs by boosting their proliferation, changing their size, and delaying their senescence. It is strongly in favor of clinical dose manufacturing. Both autologous and allogeneic human serum has been previously used for MSCs culture with diverse results [35, 38, 39]. These may be due to batch-to-batch variation of autologous and single donor allogenic HS. Therefore, the use of pooled HS is recommended. In this study, a commercial pooled HS was used, leading to more consistent results. The immunocytochemistry results showed that Nestin expression was considerably higher in the HS group (Fig. 3D). Nestin is a type VI intermediate filament protein expressed in many cells in the early stages of fetal development and may represent a specific group of multipotent precursor cells. Studies on damaged cells have shown that Nestin expression plays a crucial role in their repair and regeneration process [40]. Moreover, recent studies have indicated that Nestin-positive cells, with MSCs characteristics, resided in pancreatic islets and could differentiate into IPCs and islet-like cell clusters [41]. Interestingly, Nestin expression was observed in most FPMSCs cultured in FBS in previous studies. Only one study reported low expression of Nestin in FPMSCs [42]. Like our study, they had derived FPMSCs from pancreatic islet-like clusters. It suggests that the source and method of FPMSCs isolation may affect Nestin expression. The in vitro manipulation and expansion of cells raise numerous concerns, including the possibility of tumor formation or genomic alterations. We evaluate the tumorigenicity potential of in vitro cultured FPMSCs by G-banding karyotyping and soft agar colony formation at various passages. The results did not show colony formation or karyotype instability in both groups. Furthermore, no tumor formation was seen up to 4 months after the transplantation of FPMSCs cultured in HS-supplemented media. The current study is the first report on establishing a xeno-free/GMP-compliant protocol for FPMSCs’ expansion . Despite that, our study had some limitations, which should be considered in future experiments. To harvest the pancreas, we used donated fetuses that had been legally aborted. The aborted fetuses were rejected in many cases because of chromosomal abnormalities, long ischemic time, and damaged bodies/tissues. Consequently, we could not find appropriate donors below 14 weeks. On the other hand, we had legal and ethical limitations in using the fetuses in the third trimester. The results of our study could have been more conclusive if we had been able to examine FPMSCs derived from the first and the third trimesters. Considering the fetal pancreas shortage and to minimize the risk of failure, we used our previously established protocol for FPMSCs derivation. In that protocol, FBS was used as a serum supplementation. Similar to the previous studies, we adapted FPMSCs to the new serum (HS) for three consecutive passages, and then a side-by-side comparison was made. We can completely omit FBS from our protocol in future studies regarding the positive effect of HS supplementation on FPMSCs’ expansion. As FPMSCs could be a potential source for beta cell generation, future studies should focus on HS’s effect(s) on FPMSCs differentiation to IPCs.

## Conclusions

Considering the results of this study, we can conclude that HS is a suitable FBS alternative for clinical-grade FPMSCs manufacturing. Supplementation of culture medium with 10% HS can yield a pure, safe, potent, and scalable population of FPMSCs. Nevertheless, more in vitro and in vivo studies are needed to complete the safety profile of FPMSCs before their clinical applications.

## Data Availability

Not applicable.

## Abbreviations

AMs: Ancillary materials
CD markers: Cluster of differentiation markers
DMEM-LG: Dulbecco’s modified eagle medium—low glucose
EDTA: Ethylenediaminetetraacetic acid
ESCs: Embryonic stem cells
FBS: Fetal bovine serum
FPMSCs: Fetal pancreatic-derived MSCs
GMP: Good manufacturing practice
HLA-DR: Human leukocyte antigen—DR isotype
HS: Human serum
ICC: Immunocytochemistry
IPCs: Insulin-producing cells
MSCs: Mesenchymal stem cells
PBS: Phosphate-buffered solution
PDT: Population doubling time
RT: Room temperature
SCs: Stem cells
T1DM: Type 1 diabetes mellitus
X-gal: 5-Bromo-4-chloro-3-indolyl β-D-galactopyranoside
